# A Case of Extraskeletal Ewing Sarcoma Treated With Wide Local Excision With Latissimus Dorsi Flap and Systemic Therapy

**DOI:** 10.7759/cureus.36175

**Published:** 2023-03-15

**Authors:** Vaidehi Mendpara, Anam Sayed Mushir Ali, Tamara Tango, Ritik Bhadana, Vaishnavi Kanisetti, Utkal Tiwari, Sweta Sahu, Mukesh Pancholi

**Affiliations:** 1 Medicine and Surgery, Government Medical College Surat, Surat, IND; 2 General Surgery, Indian Institute of Medical Science and Research, Aurangabad, IND; 3 Neurosurgery/Neurology/Internal Medicine/General Surgery, Faculty of Medicine Universitas Indonesia, Jakarta, IDN; 4 Cardiology, Yatharth Hospital, Greater Noida, IND; 5 Surgery, Bhaskar Medical College, Hyderabad, IND; 6 Surgical Oncology, Shyam Shah Medical College, Rewa, IND; 7 Surgery, Jagadguru Jayadeva Murugarajendra (JJM) Medical College, Davanagere, IND; 8 Surgery, Government Medical College Surat, Surat, IND

**Keywords:** management, latissimus dorsi flap, systemic therapy, wide local excision, extraskeletal ewing sarcoma

## Abstract

Extraskeletal Ewing sarcoma (EES) is a rare tumor of the soft tissue that looks the same as skeletal Ewing sarcoma (ES). A male in his 50s was diagnosed with extraskeletal Ewing sarcoma (EES) of the right shoulder, which had infiltrated the muscles around the shoulder joints. Although uncommon, all members of the ES family of tumors, including EES, were treated following the same general protocol for sarcoma tumors. Due to the significant tumor size in this patient and local invasion, wide local excision and a latissimus dorsi flap were required. This case highlighted the management of EES, including the surgical removal of the mass on the right shoulder, followed by chemotherapy, which led to a successful outcome.

## Introduction

The Ewing sarcoma family of tumors (ESFT) includes malignant small blue round cell tumors (SBRCT) such as Ewing sarcoma (ES), extraskeletal Ewing sarcoma (EES), peripheral primitive neuroectodermal tumor (pPNET), and Askin tumor [1]. The grouping of these tumors is due to the same site of origin, mesenchymal progenitor cells, and they share the same genetic predisposition resulting from chromosomal translocations t(11;22)(q24;q12) and t(21;12)(22;12), which lead to the formation of abnormal transcription factors such as EWS-FLI-1 and EWS-ERG, respectively [2]. Ewing sarcoma is the second most common primary malignant bone tumor affecting children and adolescents after osteosarcoma [3]. Showing a mild but increased predominance in white males, this tumor usually affects the diaphysis of the bones of the lower extremities and the pelvis [4]. EES, however, shows a bimodal age distribution without any specific racial or gender predilection and usually affects the axial skeleton in the paravertebral areas, as opposed to ES, which usually affects the appendicular skeleton [5,6]. Magnetic resonance imaging (MRI) is a modality to detect and stage diseases. Since these diseases can aggressively metastasize, a fluorodeoxyglucose positron emission tomography (FDG PET) scan is typically used to detect the metastases. The treatment of these tumors follows the National Comprehensive Cancer Network (NCCN) and the European Society for Medical Oncology guidelines, where all members of the ESFT are typically treated locally with excision or radiation followed by multiple rounds of chemotherapy with varying combinations of chemotherapeutic agents [7].

## Case presentation

**Figure 1:**
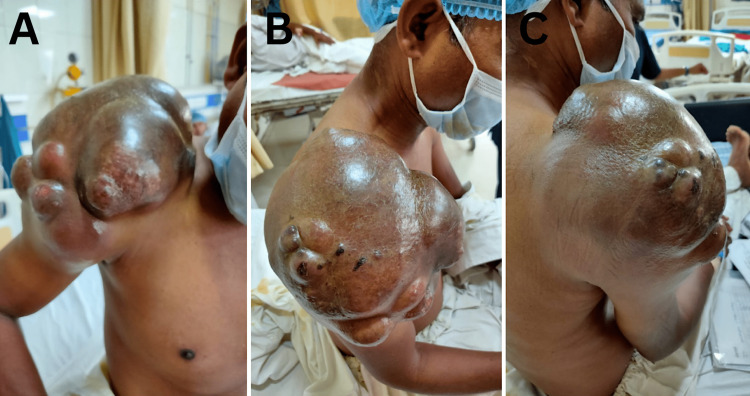
Clinical picture of a largely multi-loculated mass with the size of approximately 17.6 × 16.9 × 22 cm located in the right shoulder region A-C: View from the front, side, and back, respectively

A male in his 50s came to the doctor with right shoulder swelling that had been going on for 10 years. The patient did not experience any pain due to this gradually developing mass. After being examined, the lump was painless, non-tender, and multi-lobulated (Figure 1). During palpation, the mass was found to be fixed. The ultrasonography (USG) of the local portion showed a massive ill-defined lobulated heterogeneous echotexture lesion with low internal vascularity along with the involvement of the right shoulder. This lesion infiltrated the underlying muscles around the shoulder joint. The bony cortex that lies beneath the skin appeared to be healthy. It was hypothesized that a malignant neoplastic etiology was responsible for it, and a sarcomatous lesion was most likely the cause.

On June 15, 2021, an MRI of the right shoulder showed a relatively well-defined, mostly multi-loculated, heterogeneous lesion measuring about 17.6 × 16.9 × 22 cm, with internal hemorrhagic areas in the tuberosity and muscle infiltration. Small subchondral cystic changes were seen at the head of the humerus near the greater trochanter, with cortical erosions in the acromion process and the spine of the scapula. The right humeral head revealed a normal contour. The right humeral head and proximal shaft demonstrated normal signal intensity. A few small right axillary lymph nodes, probably reactive lymph nodes, were also noted.

**Figure 2:**
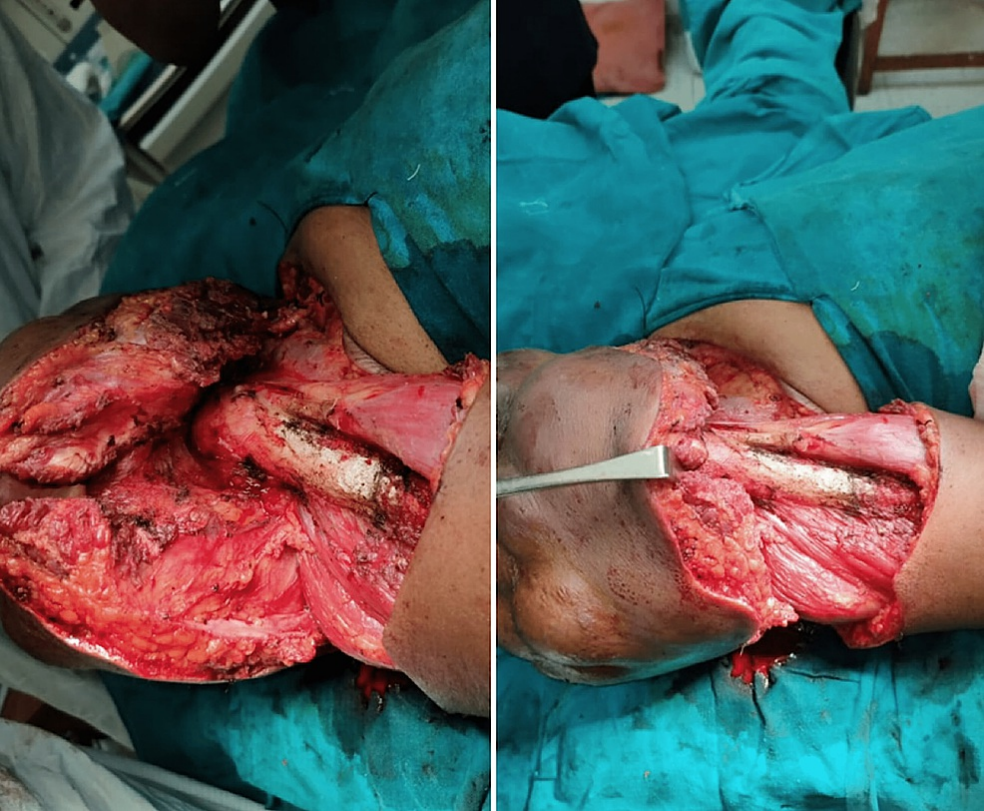
Intraoperative removal of sarcomatous mass on the right shoulder

After that, a Trucut biopsy was performed on June 24, 2021, and the results led to the possibility of two different types of sarcoma: extraskeletal soft-tissue sarcoma and fibromyxoid sarcoma. At the same time, the high-resolution computed tomography (HRCT) scan of the chest did not show any evidence of metastatic deposits. So, the patient was set to have a major surgery on July 16, 2021, which included a wide local excision, a total scapulectomy, the removal of the lateral one-third of the clavicle, and the rotation of the latissimus dorsi flap (Figure 2). The wide local excision of the tumor, weighing approximately 5 kg, was done. It led to the removal of the lateral one-third of the clavicle, the rotator cuff, and the whole scapula, separate from the humeral head, as the tumor had involved those structures. The brachial plexus, axillary artery, and vein in the proximity were preserved and were not damaged. After that, a latissimus dorsi flap with a skin flap was created with the thoracodorsal pedicle. This flap was placed along the defect after tumor excision.

The patient had given negative consent for forequarter amputation, so a mesh (drill beat mesh fixed with Ethibond number 2 {Ethibond Inc., Somerville, NJ}) was done between the remaining clavicle and humerus to aid the postoperative arm movement. The remaining defect was closed with split-thickness grafting (STG) procedure.

**Figure 3:**
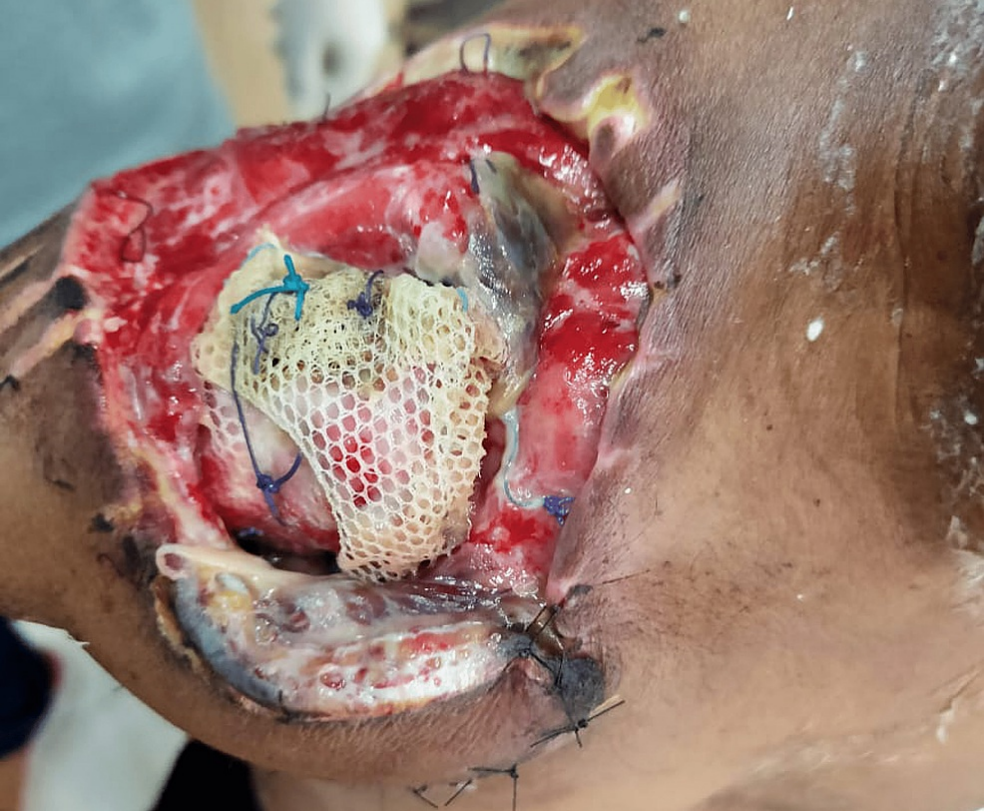
After the removal of approximately 5 kg tumor on the right shoulder

An approximately 5 kg tumor mass was successfully removed from the right shoulder (Figure 3).

**Figure 4:**
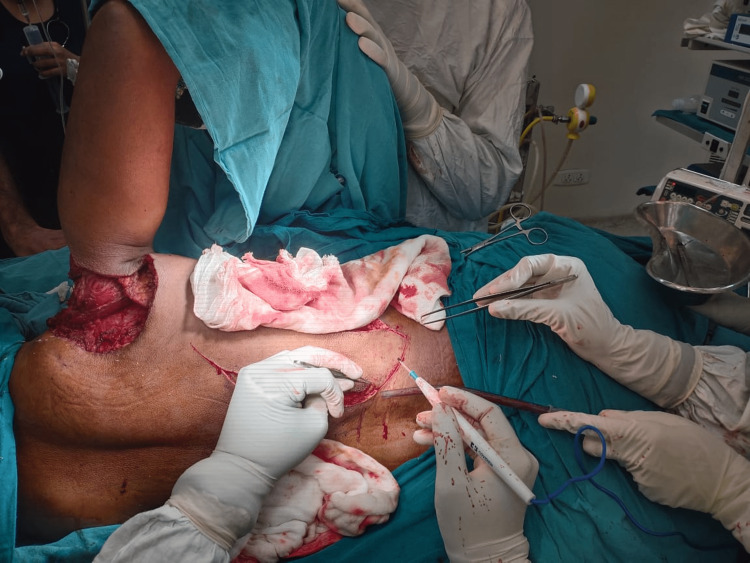
Creating a latissimus dorsi flap The latissimus dorsi flap was obtained from the right side of the patient’s back

The patient was positioned in a right lateral decubitus position, and the procedure to generate a latissimus dorsi flap was performed (Figure 4).

**Figure 5:**
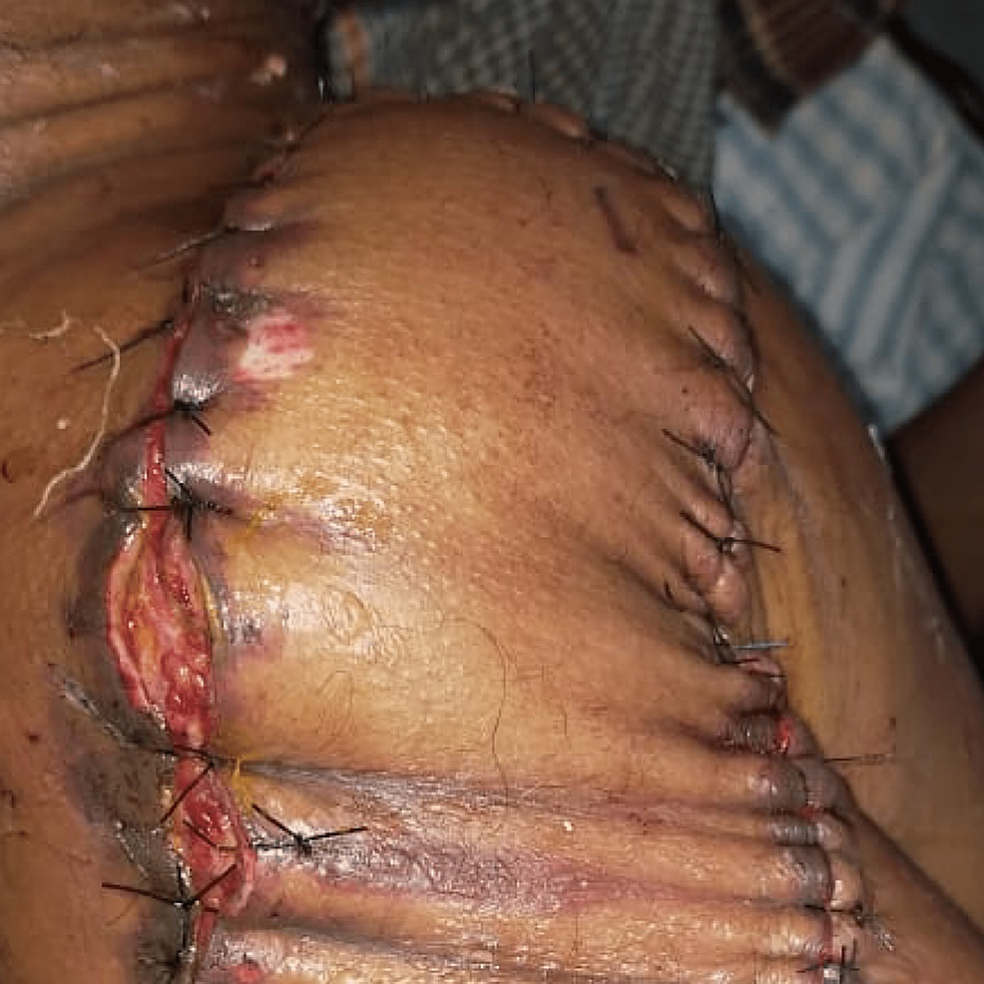
Postoperative latissimus dorsi flap on the right shoulder

After that, the latissimus dorsi flap was sewn into the right shoulder (Figure 5).

**Figure 6:**
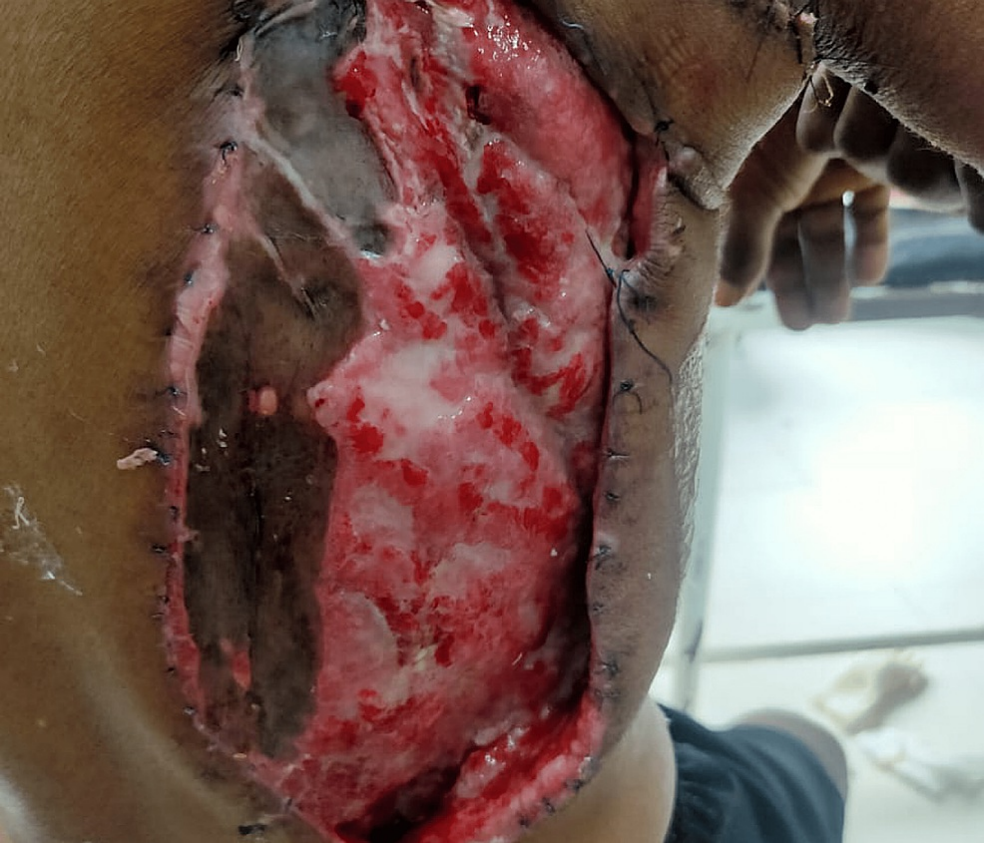
The wound at the latissimus dorsi site before the skin grafting procedure

The wound at the latissimus dorsi site before the skin grafting procedure is depicted in the image below (Figure 6).

**Figure 7:**
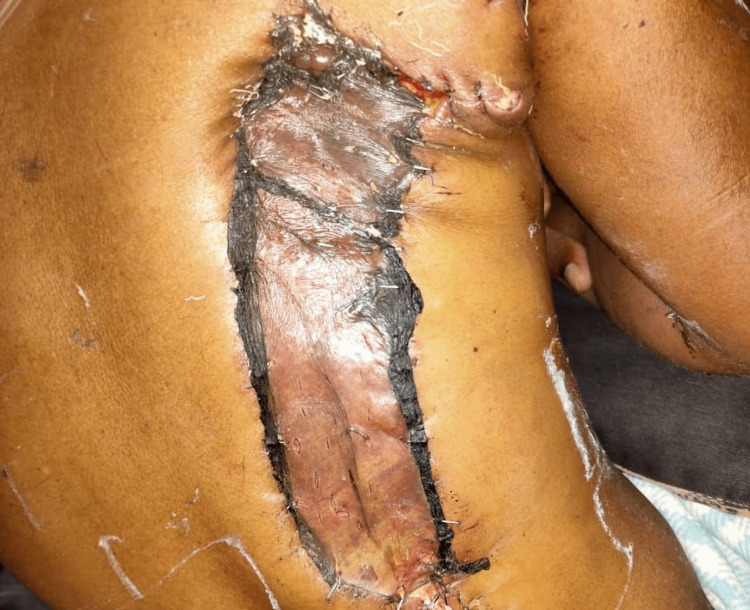
Clinical picture of the right side of the patient’s back showing postoperative skin grafting on the latissimus dorsi donor site

A skin grafting procedure was later conducted using the skin from the donor’s thigh. This skin graft was carefully placed on the latissimus dorsi donor site (Figure 7).

**Figure 8:**
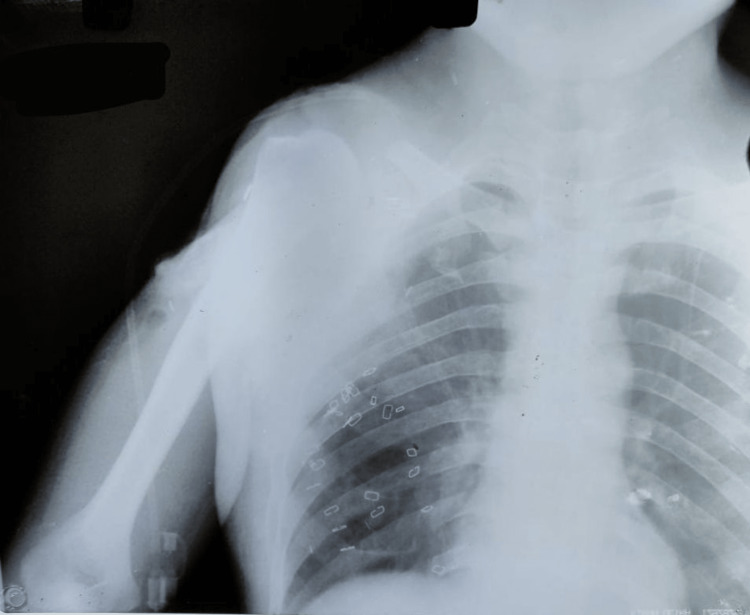
Postoperative X-ray of the right shoulder after the EES removal surgery EES: extraskeletal Ewing sarcoma

An X-ray of the right shoulder was performed after the EES removal surgery, which showed no visible mass on the right shoulder (Figure 8).

Five days after the surgery, a wound gap was found at the site of the latissimus dorsi flap. This was taken care of in a conservative way. Moreover, the histopathology on July 29, 2021, indicated that the skin margins on the superior, inferior, medial, and lateral sides were all clear of tumor. Vincristine, Adriamycin (doxorubicin), actinomycin D (dactinomycin), and cyclophosphamide were the first chemotherapy drugs administered on August 27, 2021. The chemotherapy medications were administered in a total of eight cycles over nine weeks. The postoperative movement at the shoulder was moderate.

## Discussion

Ewing sarcoma (ES) is a neoplasm that is aggressive and rare and grows quickly. It mostly affects people between the ages of 10 and 30. It constitutes 10%-15% of all bone tumors [2]. The Ewing sarcoma family of tumors includes the classic type of Ewing sarcoma of the bone, extra-osseous ES, primitive neuroectodermal tumor of the soft tissue, peripheral neuroepithelioma, Askin tumor (a malignant small cell tumor seen in the chest wall), and atypical Ewing sarcoma. All of these tumors share similar histology and immunochemistry due to their shared mesenchymal origin [8]. In extraskeletal Ewing sarcoma (EES), an important subtype of Ewing sarcoma, the most common sites include the soft tissues of the chest wall, paravertebral region, retroperitoneal space, extremities (upper thigh, buttocks, upper arm, and shoulders), and gluteal region. Other rare sites include the omentum, orbit, and skin [9]. In line with the epidemiological data, the patient in our case developed EES on the right shoulder, one of the predilection sites for EES. Moreover, our patient had also experienced a mass on the right shoulder since the previous decade, which indicated that the mass had been growing in his 40s.

Even though it has some of the same genetic features as ES, EES has different symptoms, diagnostic and treatment methods, and outcomes. It requires different treatment modalities, and the following prognosis is more favorable as well, independent of clinical factors such as age and the site of the tumor [10]. Therefore, it is important to establish an accurate diagnosis of EES, allowing suitable management of the tumor and further charting of the treatment modality. The diagnosis is usually challenging due to several factors, such as the location of the tumor, unusual clinical features, age, and variable immunohistochemistry (IHC) markers. It has no specific manifestations, clinically or radiologically. The most common symptoms include a rapidly growing, well-limited mass associated with local soft-tissue pain [11]. Other symptoms vary with the site of manifestation. In our case, the patient gradually developed a localized mass on the right shoulder without any local pain. From the physical examination, the multi-loculated mass on the right shoulder was found to be fixed, which suggested that it had adhered to the tissue beneath it.

USG, Doppler ultrasound, and computed tomography (CT) imaging of EES reveal a large, heterogeneous mass and intratumoral flow signals with a variable density as compared to the adjacent tissue and muscle and hypodense foci often due to intratumoral necrosis. The status of bone marrow cavity involvement is determined by MRI [12]. The USG of the right shoulder of this patient demonstrated an ill-defined, lobulated, heterogeneous, echotexture mass with low internal vascularity and infiltration of underlying muscle around the shoulder joint. In addition, the MRI of the right shoulder showed a 17.6 × 16.9 × 22 cm multi-loculated lesion with internal hemorrhagic areas in the tuberosity, muscle infiltration, and small subchondral cystic changes in the humerus head near the greater trochanter, cortical erosion in the acromion process, and spine of the scapula. Doppler ultrasound and CT imaging of this patient were not performed. Clinical and morphological features, CT-guided core needle biopsy, immunohistochemistry (FL11 and CD99), fluorescent in situ hybridization (FISH), and reverse transcription-polymerase chain reaction (RT-PCR) that analyze the resected specimen during the procedure are all part of the final diagnosis [8,13]. According to a molecular genetic study by Pinto et al. [8], translocations (t(11;22)(q24;q12)) are found to be positive in 80%-95% of Ewing sarcoma cases. A combination of all the mentioned techniques enhances the possibility of an accurate diagnosis. Additionally, next-generation sequencing (NGS) of soft-tissue Ewing sarcoma has been found to have higher sensitivity and specificity [14]. However, the main drawback of these molecular techniques is that they are costly or may not be available for regular testing. In our case, the patient underwent a Trucut biopsy, which resulted in two possible diagnoses (extraskeletal soft-tissue sarcoma and fibromyxoid sarcoma). Other supporting examinations, such as immunohistochemistry, FISH, and RT-PCR, were not performed due to financial issues.

Differential diagnoses based on radiology and pathology include other tumors of the Ewing sarcoma family, such as small blue round cell tumors (SBRCT), liposarcoma, and rhabdomyosarcoma [15]. The overlapping pathological, clinical, genetic, immunohistologic, and cytogenetic features present a significant diagnostic challenge to the physician. A specific staging system does not yet exist due to the rarity of EES. As a result, the American Joint Committee on Cancer (AJCC) system is recommended for all soft-tissue sarcomas. However, this system is based on lymph node metastases, which are uncommon in EES [16]. Furthermore, EES is typically diagnosed at an advanced stage [16,17]. For these reasons, Ludwig [17] proposed an alternative approach that involves staging patients based on whether they have resectable or unresectable disease while also recognizing that all patients have micrometastatic disease.

Because EES is a complex tumor to treat, a multimodal approach is essential. Deciding the combination of treatments needs to be based on the staging of the tumor both locally and systemically. Treatment guidelines have been generated by the National Comprehensive Cancer Network (NCCN) for Ewing sarcoma and various other bone cancers. These guidelines can be used to formulate treatment plans for any member of the Ewing sarcoma family of tumors. The recommended treatment modalities include radiotherapy and/or surgery along with chemotherapy [18,19]. The patient in our case had a stage 3B soft-tissue tumor in his right shoulder; therefore, he underwent surgery followed by systemic therapy. Studies have suggested that surgery (complete resection) plays a more important role in EES as compared to the skeletal type and therefore proves to be the gold standard of treatment [20,21]. For the chemotherapy regimens, systemic therapy was administered to this patient, including vincristine, Adriamycin (doxorubicin), actinomycin D (dactinomycin), and cyclophosphamide. Recent studies have proven aggressive induction chemotherapy with alternating vincristine-doxorubicin-cyclophosphamide and ifosfamide-etoposide (VDC/IE) cycles to hold a pivotal role in improving survival rates and reducing local recurrence rates [22].

## Conclusions

As demonstrated in this case report, extraskeletal Ewing sarcoma (also known as EES) is a malignant tumor that is both rare and severe. EES can affect adults. Because the clinical signs of EES might be confused with those of other diseases, it is essential to establish an accurate diagnosis by employing a variety of techniques, which include ultrasound, magnetic resonance imaging (MRI), and Trucut biopsy. The National Comprehensive Cancer Network (NCCN) protocol formulated for Ewing sarcoma serves as the basis for the treatment plan for EES. In our case, the sarcoma was in stage 3B and therefore was treated with a combination of surgical procedures and systemic chemotherapy. It was necessary to cover the significant deformity created after excision with a latissimus dorsi flap. It is absolutely necessary for us to educate people about this extremely rare malignancy.
